# Attitudes Surrounding Music of Patients With Anorexia Nervosa: A Survey-Based Mixed-Methods Analysis

**DOI:** 10.3389/fpsyt.2021.639202

**Published:** 2021-06-02

**Authors:** Aishwarya Krishna Priya, Briana Applewhite, Katie Au, Oyenike Oyeleye, Emma Walton, Caroline Norton, Olivia Patsalos, Valentina Cardi, Hubertus Himmerich

**Affiliations:** ^1^Department of Psychological Medicine, Institute of Psychiatry, Psychology and Neuroscience, King's College London, London, United Kingdom; ^2^South London and Maudsley National Health Service (NHS) Foundation Trust, London, United Kingdom

**Keywords:** music, music therapy, eating disorders, anorexia nervosa, mixed-methods research

## Abstract

Anorexia nervosa (AN) is one of the main eating disorders. It has the highest mortality of all psychiatric disorders, and the success rates of current therapies are not fully satisfactory. Thus, there is a need for novel interventions. We investigated the attitudes surrounding music of 41 patients with clinically-diagnosed AN as well as their thoughts on the potential therapeutic uses of music using a questionnaire of 50 questions. Free text responses were qualitatively analyzed for reoccurring themes with NVivo 12 software. Yes/no questions and questions of best fit were analyzed using the IBM SPSS Statistics version 25. The most prevalent theme was the positive effect of music. Most patients reported that listening to music evokes varying emotions in them (83%) which may be of positive or negative nature. Similarly, patients associated certain music with particular positive, but also with particular negative memories. A majority of patients stated that music helps to distract them (85%), helps with loneliness (59%) and helps them feel more connected to others (58%). This data indicates that people with AN make nonclinical use of music which seems to elicit positive as well as negative emotions and memories. Patients felt music is beneficial with regard to important aspects of AN, such as emotional problems, loneliness, and relationship difficulties. Most of them would also like to attend music therapy.

## Introduction

Eating disorders (EDs) are characterized by an overvaluation of weight and shape, and an unhealthy fixation or attitude toward food. According to the Diagnostic and Statistical Manual of Mental Disorders, Fifth Edition (DSM-5) there are six feeding and eating disorders: anorexia nervosa (AN), bulimia nervosa (BN), binge eating disorder (BED), avoidant restrictive food intake disorder (ARFIS), pica and rumination (1). The prevalence of EDs is around 6.4% in adults and their mortality rate is high (2). Patients suffering from AN have the highest rate of mortality of any psychiatric disorder. For example, The Standard Mortality Ratio (SMR) for people with AN for Wales and England is ~5.21 (3). In AN, the mean lifetime prevalence is ~1% in women and ~0.2% in men (2).

The main diagnostic criteria of AN according to DSM-5 are a significantly low body weight, an intense fear of weight gain and a disturbed body perception (1). Further important problems people with AN face are related to emotion processing and regulation, such as poor emotional awareness, clarity, as well as avoidance (4), in addition to interpersonal relationships and feelings of loneliness (5). Both areas have been suggested to play a key part in the development and maintenance of AN (4, 5).

According to the National Institute for Health and Care Excellence (NICE), treatment of EDs in the United Kingdom (UK) should include psychological therapy, diet counseling, and physical health monitoring. Recommended psychological therapies for adult patients with AN are Cognitive Behavioral Therapy (CBT), Maudsley Anorexia Nervosa Treatment for Adults (MANTRA), Specialist Supportive Clinical Management (SSCM), and Eating-Disorder-Focused Focal Psychodynamic Therapy (FPT). Additional therapies comprise of family therapy and occupational therapy (6). Similar recommendations are in place in other European countries. Nonetheless, a recent study showed that real-world patients with AN have a recovery rate of only 30% after 10 years and 60% after 20 years (7).

Given the low rate of recovery, there is a clear need for additional treatments. In a recently conducted systematic review on the use of music in patients with EDs which encompassed 16 studies and 3,792 participants, the use of music was found potentially beneficial as an adjunct to current treatments (8). For example, inpatients who listened to classical music during mealtimes improved food consumption (9), and vodcasts including soothing music favorably influenced eating behaviors in AN (10). However, music is presented and consumed in a variety of different ways, and not all the ways music is consumed seem to be helpful. For example, watching music videos has been reported to reinforce body dissatisfaction, a drive for thinness, bodyweight concerns, and a preoccupation with physical appearance (8, 11, 12).

In a recently conducted preparatory qualitative study to obtain input to design a structured questionnaire for the current survey, we found that patients with AN mainly associate positive memories and emotions with music (13). The aim of the current research was to further examine the attitudes of people with AN surrounding their use of music, positive and negative effects of music, and their thoughts on music as an adjunct to their treatment. We hypothesized that patients with AN would associate music mainly with positive memories and emotions.

## Methods

### Study Design

To evaluate patients' attitudes surrounding music and music therapy, we developed a questionnaire based on a preparatory study (13). The questionnaire comprised of 50 questions split into seven sections: Section one and two provided patient information, such as age, sex, and duration of illness. The third section included questions focused on participants current situation with AN. Questions in this section asked, for example, whether participants felt unwell with anorexia, found eating difficult and experienced anxieties around food and their look. The purpose of these section within the questionnaire is to collect clinical characteristics of the study sample. The fourth section required participants to answer questions on the time spent listening or watching music. The fifth section asks about music-making and how participants engage with music. The sixth section asks about their experiences with the therapeutic uses of music, and the final section is about patient perspectives of music and music therapy. Questions were a mix of short answer responses, “yes/no” questions, as well as choosing a statement that best fits from “strongly agree” to “strongly disagree.” The questionnaire was distributed to patients between May and June 2020. The questionnaire is depicted as Supplementary Material.

### Participants

Forty-one patients (40 females and 1 male) who had been clinically diagnosed by a psychiatrist as having AN were recruited within the South London and Maudsley NHS Trust (SLaM) eating disorder inpatient and outpatient services. Participants were given a written explanation of the nature of this study as well as the confidentiality maintained throughout their participation. They gave written informed consent before entering the study and then completed the questionnaire. The study was approved as an audit by the South London and Maudsley NHS Foundation Trust (SLaM).

Study participants were between 18 and 65 years old; mean age 33.17 ± 12.90 standard deviation (SD). Fourteen (34%) patients were treated as outpatients, eight (20%) were inpatients, five (12%) were treated by their Community Mental Health Team (CMHT), four (10%) patients were treated in the day-care service, nine (22%) patients were not receiving any form of treatment, and one (2%) patient was seeking private psychological care. Duration of illness was between one and a half and 50 years (mean: 12.43 ± 11.30 SD). Of the 41 patients, 30 (73%) patients reported being currently unwell with AN, 30 (73%) finding eating difficult, 38 (93%) having anxieties around food, and 35 (85%) being worried about the way they look. However, 23 (56%) stated that they were currently coping with their AN, and two (5%) reported that they had overcome their AN.

### Data Analyses

The questionnaires were analyzed with IBM SPSS Statistics version 25 for descriptive statistics and NVivo 12 software for qualitative statistics on the free-text responses. The descriptive statistics comprised the number and the percentage of people within the whole sample (*N* = 41) who gave a specific answer. Using content analysis, Briana Applewhite extracted the themes for the qualitative statistics which were subsequently agreed with Aishwarya Krishna Priya and Hubertus Himmerich.

## Results

### Qualitative Analysis of Free-Text Answers

The mean number of words written by participants in the free-text responses was 104.61; the median was 65. The minimum number of words to answer the free-text questions was 7, the maximum 1,036.

Following the analysis of the free-text responses, 16 total themes were obtained from the data collected. The themes extracted (the number of questionnaires they appeared in/the number of references made) are as follows: *Benefit of Music* (20/31), *Benefit of Music Therapy* (3/4), *Frequency* (1/1), *How Music Makes You Feel (*14/18), *Importance of Music* (9/11), *Interest in Music Therapy* (22/22), *Music Making* (9/14), *Music Preference* (9/13), *Negative Effects of Music* (22/28), *Negative Emotion Elicited* (21/30), *Negative Memory Association* (14/19), *Neutral Emotion Elicited* (1/1), *Neutral Memory Association* (1/1), *Positive Effect of Music* (38/59), *Positive Emotion Elicited* (22/28), and *Positive Memory Association* (12/24).

The most prevalent themes coded throughout the participants' text were *Positive Effects of Music, Benefit of Music, Negative Emotion Elicited, Negative Effect of Music*, and *Positive Emotion Elicited*. Based on the data collected, six theme groups were created: *Positives, Negatives, Neutrals, Preference* (of music consumption), *Beliefs*, and *Music Therapy*. The *Positives* grouping accounted for 36.5% of the total themes coded, while Negatives and Beliefs made up 25.3 and 19.7% of the total references, respectively (Table 1).

**Table 1:** Top 10 most frequently used words in free-text responses.

**Word**	**Length**	**Count**	**Weighted percentage**	**Similar words**
Music	5	654	6.84%	Music, musical, musicals
Helps	5	285	2.98%	Help, helped, helpful, helpfully, helping, helps
Feel	4	216	2.26%	Feel, feeling, feelings, feels
Listening	9	128	1.34%	Listen, listened, listening
Negative	8	122	1.28%	Negative, negatively, negatives
Songs	5	117	1.22%	Song, songs
Used	4	117	1.22%	Use, used, useful, using
Make	4	116	1.21%	Make, makes, making
Research	8	112	1.17%	Research
Distraction	11	103	1.08%	Distract, distracted, distracting, distraction, distracts

Throughout all of the free-text responses from the 41 participants, the most commonly referenced theme was the positives associated with music, with the specific themes of *Positive Effect of Music* (59 references), *Positive Emotion Elicited* (28 references), as well as *Positive Memory Association* (24 references) totaling 111 references. Participants described their interactions with music as generally positive and an experience that allows them to deal with difficult emotions with a more productive outlet, while also serving as a distraction from their thoughts surrounding their eating disorder. One patient stated, “I enjoy listening to music and finding new music… it's quite a good way of keeping my mind occupied so I'm not thinking negatively about myself.” While another stated, “[music] has become an absolute necessity in my daily life and there is not one day in which I don't listen to music. It helps me deal with my emotions and feelings, especially when I can relate to the music.”

Many participants described positive memories that they have associated with music. One patient recalled a particularly positive memory of a music group for patients with AN, stating, “…the music group gave me something to enjoy, be distracted by and focus on, especially as I find concentration difficult. It also gave me something to commit too when I struggle with motivation especially when we were preparing for a performance. It was good for bonding with others on the ward especially when we performed together.”

The second-largest coded theme grouping were *Negatives* which accounted for 77 coded references in total. While there were many positive effects of music that participants explicated, there were also quite a few negatives. When asked in the free-text answers if they could think of any negative effects that music has, participants described that often, when listening to something sad or melancholy, this can trigger this same emotion within themselves, causing them to mirror the emotions heard in the music. One participant said, “If the emotion of the song is negative this is passed on to me. Sometimes I'll listen to sad pieces, this feeling can then last a few hours.” Another stated, “…listening to sad or very mellow music can make me feel more low/anxious.”

Participants also described negative memories that they associate with specific songs and how these influenced negative emotions within themselves. A patient stated, “It can remind me of difficult times (e.g., certain songs remind me of times I was obsessive about exercise and listened to them a lot while exercising).” While some music can serve as a positive distraction for some, for others, it produces the opposite effect. “When on the ward…music was often put on during meals as a so-called ‘distraction' and sometimes I would end up unable to eat because the song playing was making me feel so helpless and depressed.”

**Figure 1:**
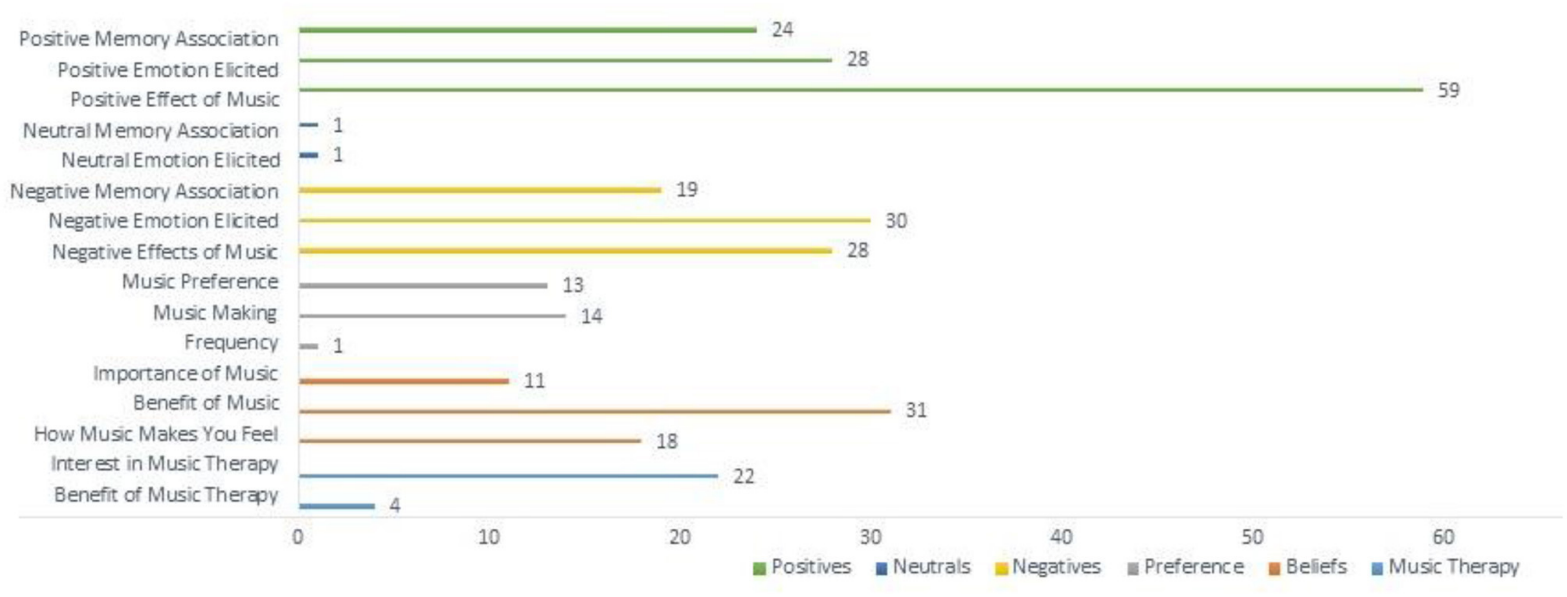
Distribution of the frequency of themes coded from all 41 participants.

*Beliefs* accounted for the third-largest theme group with 60 total coded references. This theme highlights when participants described the importance of music in their lives, the benefit they see to music, as well as how music makes them feel (Figure 1). Music Preference (listening, music-making, and performance) as well as the frequency of music listening, accounted for 9.2% of the coded data. Participants listed genres of music they listen to, their music likes and dislikes, including where and how they consume the music they listen to. Neutral themes (*Neutral Memory Association* and *Neutral Memory Elicited*) only accounted for 0.65% of the total data, suggesting that most people feel strong positive or negative emotions surrounding music, and hardly feel indifferent about the subject.

Regarding the *Music Therapy* theme grouping, there was a total of 26 coded references throughout the free-text data. The specific themes cited were the *Benefits of Music Therapy* as well as *Interest in Music Therapy*. Patients seemed to have a general interest in learning more about music therapy and how it could be potentially beneficial as an adjunct to their current treatment. A few patients had experience with music therapy and even participated in music therapy sessions. However, most had never had any involvement with it. Some patients gave suggestions on how music could be implemented to their treatment, specifically in inpatient units.

One patient expressed her interest stating, “I think music therapy should be offered as an option in all EDUs. All research is good. If there's potential for music therapy to help, then it should be investigated.” Overall, the topic of music brought about passionate perspectives. Whether positive or negative, participants seemed eager to share their experiences with music as well as being interested in learning more about music therapy and participating in sessions along with their current therapies.

### Quantitative Analysis

The mean duration in hours patients reported listening to music while doing other things was 3.57 (±4.88) per day, and they stated spending a mean of 1.33 (±3.43) listening to music as their main activity. Regarding their experience with music, most patients reported that they had learned to sing or to play an instrument (71%) and that they sing or play an instrument on a regular basis (61%). In terms of their experience with the therapeutic use of music, more than half of the patients specified that they use music to cope with difficult emotions (58%), as a distraction from their thoughts (78%), or as a distraction from their feelings (76%). Even though 78% stated that they would like to attend a music therapy session, only 20% reported that they had ever attended a music therapy session, and only 5% said that their psychiatrist or psychotherapist has ever used music during their treatment sessions. For more detailed information on patients' experience with music or music therapy (see Table 2).

**Table 2:** Answers to dichotomous questions regarding participants' experience with music and the therapeutic use of music.

	**Yes**	**No**	**N/A**
**Experience with music**			
I have learned to sing or to play an instrument	29 (71%)	12 (29%)	0 (0%)
I regularly sing or play music	25 (61%)	16 (39%)	0 (0%)
I make music with others (friends, family members, in a band or orchestra)	8 (20%)	33 (80%)	0 (%)
I regularly record, mix or produce music	1 (2%)	40 (98%)	0 (0%)
I perform music on a regular basis	3 (7%)	38 (93%)	0 (0%)
I have always wanted to try or learn to play an instrument	24 (59%)	16 (39%)	1 (2%)
**Experience with the therapeutic use of music**			
I use music to cope with problems	19 (46%)	20 (49%)	2 (5%)
I use music to cope with difficult emotions	24 (58%)	17 (42%)	0 (0%)
I use music as a distraction from my thoughts	36 (78%)	5 (12%)	0 (0%)
I use music as a distraction from my feelings	31 (76%)	9 (22%)	1 (2%)
I use music to help manage my anorexia	17 (42%)	24 (58%)	0 (0%)
I would like to attend a music therapy session	32 (78%)	8 (20%)	1 (2%)
I have attended a music therapy session	8 (20%)	33 (80%)	0 (0%)
My psychiatrist/psychotherapist has used music during my treatment sessions	2 (5%)	39 (95%)	0 (0%)

*The numbers in the table refer to the number of people who gave this specific answer; the % value refers to the proportion of the whole sample of N = 41. All answers not given or not clearly marked counted as “N/A.”*

A majority of patients agreed or strongly agreed that listening to music evoked varying emotions in them (83%), and that they felt happy and more positive when listening to music (63%). However, 36% agreed or strongly agreed that they felt more negatively and 10% stated they felt sad when listening to music. Both positive as well as negative memories were associated with certain music as 86% agreed or strongly agreed to associate certain music with a particular positive memory in their life, and 71% agreed or strongly agreed to associate certain music with a particular negative memory in their life. A majority of patients agreed or strongly agreed with the statements that listening to music helps to distract them (85%), helps with loneliness (59%), and helps them feel more connected to others (58%). But only a minority of patients found that music would help with the anorexic voice (34%), help to concentrate (32%), help to eat (25%), or help with weight and shape concerns (22%).

Seventeen percent agreed or strongly agreed that watching music videos would make their weight and shape concerns worse, 37% were neutral about this statement, while 41% disagreed or strongly disagreed with this statement. Thirty percent agreed or strongly agreed that watching music videos was fun, 24% were neutral about it and 41% disagreed or strongly disagreed with this statement.

Seventy-three percent of participants strongly agreed that there should be more research on music in AN, and 71% strongly agreed that they would take part in research on music therapy for people with AN. For more detailed information on the quantitative outcome of our survey (see Table 3).

**Table 3:** Questions and answers regarding patients' perspective of music and music therapy.

	**Strongly agree**	**Agree**	**Neutral**	**Disagree**	**Strongly disagree**	**N/A**
Listening to music evokes varying emotions in me	18 (44%)	16 (39%)	3 (7%)	4 (10%)	0 (0%)	0 (0%)
I feel happy when listening to music	7 (17%)	19 (46%)	13 (32%)	0 (0%)	2 (4.9%)	0 (0%)
When I listen to music, I generally feel more positive	7 (17%)	25 (61%)	7 (17%)	0 (0%)	1 (2%)	1 (2%)
I feel sad when listening to music	1 (2%)	14 (34%)	18 (44)	5 (12%)	1 (2%)	2 (5%)
When I listen to music, I generally feel more negative	2 (5%)	2 (5%)	7 (17%)	24 (59%)	6 (15%)	0 (0%)
I associate certain music with a particular positive memory in my life	19 (46%)	15 (37%)	2 (5%)	3 (7%)	2 (5%)	0 (0%)
I associate certain music with a particular negative memory in my life	13 (32%)	16 (39%)	7 (17%)	3 (7%)	2 (5%)	0 (0%)
Listening to music helps distract me	9 (22%)	26 (63%)	3 (7%)	0 (0%)	3 (7%)	0 (0%)
Music helps with the anorexic voice	5 (12%)	9 (22%)	16 (39%)	7 (17%)	4 (10%)	0 (0%)
Music helps me to concentrate	4 (10%)	9 (22%)	12 (29%)	10 (24%)	6 (15%)	0 (0%)
Music helps me to eat	2 (5%)	8 (20%)	10 (24%)	13 (32%)	7 (17%)	1 (2%)
Music helps with weight and shape concerns	3 (7%)	6 (15%)	8 (20%)	12 (29%)	12 (29%)	0 (0%)
Music helps with loneliness	6 (15%)	18 (44%)	8 (20%)	3 (7%)	6 (15%)	0 (0%)
Music helps me feel more connected to others	10 (24%)	14 (34%)	9 (22%)	3 (7%)	5 (12%)	0 (0%)
Watching music videos makes my weight and shape concerns worse	2 (5%)	5 (12%)	15 (37%)	12 (29%)	5 (12%)	2 (5%)
Watching music videos is fun	4 (10%)	8 (20%)	10 (24%)	12 (29%)	5 (12%)	2 (5%)
There should be more research on music in anorexia nervosa	9 (22%)	19 (46%)	6 (15%)	3 (7%)	2 (5%)	2 (5%)
There should be more research on music therapy for people with anorexia nervosa	11 (27%)	19 (46%)	7 (17%)	1 (2%)	2 (5%)	1 (2%)
I would take part in a trial to assist in research into music therapy for anorexia nervosa	14 (34%)	15 (37%)	4 (10%)	3 (7%)	4 (10%)	1 (2%)

## Discussion

We conducted a mixed-methods study consisting of a qualitative and quantitative analysis of the views of patients with AN have concerning music, the positive and negative effects music can produce in their lives as well as their interest in music therapy as an adjunct to their current treatment. The study sample included patients in different treatment settings (specialist inpatient, daycare, and outpatients as well as community mental health services and private psychotherapy practices). Most patients were currently unwell with AN.

In the qualitative analysis, the most common theme grouping were *Positives* associated with music. The *Negatives* theme grouping accounted for 25.3% of the total coded data, with *Negative Emotion Elicited* being the most common theme. The third most common theme grouping were *Beliefs* surrounding music with the *Benefit of Music* accounting for the most coded themes within this grouping. The qualitative data suggest that participants believe that music elicits mainly positive, but also negative emotions and that there are beneficial uses of music for their recovery.

The main results of the quantitative analysis were that most patients had learned to sing or play an instrument and do this on a consistent basis. Furthermore, most patients reported using music to cope with difficult emotions and as a distraction from their thoughts and feelings. Seventy-eight percent were interested in attending a music therapy session. These findings indicate that most patients with AN have a prior background in engaging with music and music making, and have a strong interest in participating in music therapy. They also believe that music can help them with difficult emotions, thoughts, and feelings.

In addition, patients reported that listening to music evoked varying emotions and memories within them, although mainly positive. A majority of patients stated that listening to music helps to distract them, helps with feelings of loneliness and allows them me feel more connected to others. Only a minority of patients found that music would help with nonengagement of the anorexic voice, assist with concentration, improve eating patterns or help with weight and shape concerns. This means that according to patients with AN, music might help with significant AN-related emotional and interpersonal problems (4, 5) but less so with the core symptoms of AN according to DSM-5, which include a significantly low body weight, an intense fear of weight gain and a disturbed body perception (1) as only a minority of patients found that music would help with eating or improving weight and shape concerns.

Thus, the results of this study point to a promising possibility of the ways music can be used in emotional regulation, based on patients recalling ways that music can amplify certain emotions, and serve as a distraction from negative thoughts they may be having, specifically regarding their AN. Our survey demonstrates that people with AN make nonclinical use of music and find this helpful. However, music was also perceived as eliciting not only positive, but different emotions. The type of emotions might depend on the type of music, and the individual's personal experiences, memories, and circumstances.

In previous studies on the uses of music in EDs, researchers found that listening to classical music reduced anxiety and improved consumption of food; listening to Mozart reduced body width estimation within patients with BN; listening and playing music-evoked positive emotions, memories as well a general interest amongst patients in attending music therapy sessions (8–10). These previous findings align with our current research because participants described positives effects that music has on their life as well as expressing keen interest in participating in music therapy sessions themselves.

However, in addition to our initial hypothesis that patients with AN would associate music mainly with positive memories and emotions, our study found that music has potentially negative effects as well; participants described that when already low in mood, certain music can amplify these negative emotions. Additionally, patients reported that certain songs can be linked to negative memories and emotions and could even create feelings of adversity, triggering detrimental actions that could influence eating patterns. Even though most patients did not think that watching music videos would worsen their weight and shape concerns, 17% still thought that watching music videos would make their weight and shape concerns worse, and 41% disagreed or strongly disagreed that watching music videos was fun. Thus, at least a subgroup of people with AN are concerned with certain ways music is presented, which is in line with previous studies showing a worsening of ED symptoms after watching music videos (11, 12). Our study illuminates the individual differences and relationship patients have with music, and whether a certain presentation or type of music is perceived as pleasurable.

Nonetheless, from a psychotherapeutic perspective, even negative emotions such as anger elicited by music could be therapeutically helpful, because patients with AN have difficulties with expressing these negative emotions. Recognition, regulation, and expression of emotions are deemed to be highly relevant psychotherapeutic aspects in people with EDs (14). This would enable the therapist and the patient to get access to negative emotions and work through processing them. For this reason, the effects of music should be explored in further research, namely whether music can help with access to the entirety of a patient's emotions, the effects certain genres can have on mood in patients with AN, as well as the effects music has on memory association.

Despite these interesting findings, there were some limitations. The sample size was small (*n* = 41), and the participants were ethnically homogenous with mainly Caucasian participants. There was only one male participant, the rest (40) were all females, so gender differences related to music could not be explored. This is relevant because gender differences have been described in patients with EDs (15). Due to the restricted sample size, subgroups of patients, e.g., patients with autistic traits, specific personality traits, or psychiatric comorbidities could not be analyzed separately. However, this would be clinically relevant as these subgroups pose specific diagnostic and therapeutic challenges (8, 16). We surveyed one service in London, so the results are not generalizable to the entirety of the UK or worldwide. Furthermore, we only sampled individuals with AN. As mentioned in the introduction, EDs should be viewed as a cluster of symptoms producing symptoms related to body image disturbance, disordered eating, and their physical and psychosocial consequences. Further studies should be enacted to see if our results can be reproduced in individuals with BN, BED, ARFID, or other EDs, across EDs patients of different cultural backgrounds and in male, female, and nonbinary patients.

Further directions of research could include asking psychiatrists, psychotherapists, occupational therapists, nurses, family therapists, and social workers whether they would consider adding an element of music into their therapies.

A challenge that could arise when enacting group music therapy or standardized individual music therapy is that music preference varies widely amongst individuals and perceptions of music differ even more for different people. This limitation could prove challenging for standardization of music therapy practices, and therefore may not reach a level of standardization comparable to CBT. Despite these challenges, our analysis based on the view of patients with AN showed that music might have beneficial effects and should be further explored and used during psychological therapies as a tool to elicit and modulate emotions. For further research, an encouraging finding is that 73% of patients agreed that there should be more research on music in AN, and 71% stated that they would take part in research on music therapy for people with AN, pointing to a promising potential of the novel uses music can have for those struggling with AN.

## Data Availability Statement

The completely anonymized raw data supporting the conclusions of this article will be made available by the authors upon request to the corresponding author, without undue reservation.

## Ethics Statement

Ethical review and approval was not required for the study on human participants in accordance with the local legislation and institutional requirements. The patients/participants provided their written informed consent to participate in this study.

## Author Contributions

AK, BA, VC, and HH designed the study. KA, OO, EW, CN, OP, and HH recruited the participants. AK, BA, and HH evaluated the data. AK, BA, OP, VC, and HH wrote the manuscript. All authors approved the final manuscript.

## Conflict of Interest

The authors declare that the research was conducted in the absence of any commercial or financial relationships that could be construed as a potential conflict of interest.
