# Supplementary material for: Attitudes Surrounding Music of Patients With Anorexia Nervosa: A Survey-Based Mixed-Methods Analysis
Source: Front Psychiatry. 2021 Jun 2;12:639202. doi: 10.3389/fpsyt.2021.639202 (PMC8206484; doi:10.3389/fpsyt.2021.639202)
Supplement: Supplementary file 1 [file Data_Sheet_1.pdf]

Perspective of Music and Music Therapy Questionnaire

We have created this questionnaire to help develop research around the use music in people with anorexia nervosa. Your input will help improve our therapeutic service. Please answer the questions according to what you think. Participation is voluntary and data will be anonymised and used for research and service development purposes only.

Insert your own text if required. If you have any questions about this survey, ask Dr Hubertus Himmerich: hubertus.himmerich@kcl.ac.uk. Thank you for your participation. If you have completed a digital version of this questionnaire, please send it back to hubertus.himmerich@kcl.ac.uk.

Personal Information

1.	My age		**years**
2.	My gender		**female/male/other**

3. My **current treatment**. I am currently being treated in the following service (e.g. FREED, Outpatient, Daycare, SEED, Step-up, Inpatient, Community Mental Health Team) or am currently not in treatment:

4. **Duration** of my current treatment in the above-mentioned service (if applicable):

Duration of illness:

5. For how many years have you had/did you have anorexia nervosa?

	**Years**

Current situation with my anorexia nervosa:

		**Yes**	**No**
6.	I am currently unwell with anorexia.		
7.	I am currently finding eating difficult.		
8.	I am currently experiencing anxieties around food.		
9.	I am currently worried about how I look.		
10.	I am currently coping with my anorexia.		
11.	I have overcome my anorexia.		

Please turn the page.

Time spent listening to music or watching music performances:

12. How many **hours per day** do you spend listening to music **while you are doing other things**?

	**Hours**

13. How many **hours per day** do you spend listening to music **as a main activity**?

	**hours**

Actively engaging with music. Please check one box for each question:

		**Yes**	**No**
14.	I have learned to sing or to play an instrument.		
15.	I regularly sing or play music.		
16.	I make music with others (friends, family members, in a band or orchestra).		
17.	I regularly record, mix or produce music.		
18.	I perform music on a regular basis.		
19.	I have always wanted to try or learn to play an instrument.		

20. How many **hours per week** do actively engage with music (listening or playing)?

	**Hours**

Experience with the therapeutic use of music. Please check one box for each question:

		**Yes**	**No**
21.	I use music to cope with problems.		
22.	I use music to cope with difficult emotions.		
23.	I use music as a distraction from my thoughts.		
24.	I use music as a distraction from my feelings.		
25.	I use music to help manage my anorexia.		
26.	I would like to attend a music therapy session.		
27.	I have attended a music therapy session.		
28.	My psychiatrist/psychotherapist has used music during my treatment sessions.		

Please turn the page.

Perspective of music and music therapy. Please check one box for each question:

		Strongly Disagree	Disagree	Neutral	Agree	Strongly agree
29.	Listening to music evokes varying emotions in me.					
30.	I feel happy when listening to music.					
31.	When I listen to music, I generally feel more positive.					
32.	I feel sad when listening to music.					
33.	When I listen to music, I generally feel more negative.					
34.	I associate certain music with a particular positive memory/particular positive memories in my life.					
35.	I associate certain music with a particular negative memory/particular negative memories in my life.					
36.	Listening to music helps distract me.					
37.	Music helps with the anorexic voice.					
38.	Music helps me to concentrate.					
39.	Music helps me to eat.					
40.	Music helps with weight and shape concerns.					
41.	Music helps with loneliness.					
42.	Music helps me feel more connected to others.					
43.	Watching music videos makes my weight and shape concerns worse.					
44.	Watching music videos is fun.					
45.	There should be more research on music in anorexia nervosa.					
46.	There should be more research on music therapy for people with anorexia nervosa.					
47.	I would take part in a trial to assist in research into music therapy for anorexia nervosa.					

Please turn the page.

48. In your own words, how is music beneficial or helpful for you?

49. In your own words, what negative effects does music have?

50. Do you have any further comments or suggestions for research around music in anorexia nervosa?

I agree that my data are used for service improvement and research. I am aware that my participation is voluntary and that my data will be anonymised and used for research purposes only.

Signature or initials:

Thank you for taking part in this survey and answering the questions.
